# Essential components of an effective transition from paediatric to adult neurologist care for adolescents with Duchenne muscular dystrophy; a consensus derived using the Delphi methodology in Eastern Europe, Greece and Israel

**DOI:** 10.1186/s13023-024-03270-2

**Published:** 2024-07-09

**Authors:** Maria Judit Molnar, Léna Szabó, Oana Aurelia Vladacenco, Ana Maria Cobzaru, Talya Dor, Amir Dori, Georgios Papadimas, Lenka Juříková, Ivan Litvinenko, Ivailo Tournev, Craig Dixon

**Affiliations:** 1 Director of Institute of Genomic Medicine and Rare Disorders, Semmelweis University, Budapest, Hungary; 2 Pediatric Center, Semmelweis University, Budapest, Hungary; 3 University of Medicine and Pharmacy Bucharest, Bucharest, Romania; 4 University Emergency Hospital Bucharest, Bucharest, Romania; 5 Pediatric Neurology Unit, Hadassah University Hospital, Jerusalem, Israel; 6 Sheba Medical Center at Tel-Hashomer, Neurology Clinic, Ramat-Gan, Israel; 7 First Department of Neurology, University of Athens, Athens, Greece; 8 Department of Pediatric Neurology, University Hospital Brno, Brno, Czech Republic; 9 Pediatric Neurology Department, SHATPD “Prof. Dr. Ivan Mitev”, Sofia, Bulgaria; 10 Department of Neurology, University Hospital Aleksandrovska, Medical University, Sofia, Bulgaria; 11 Department of Cognitive Science and Psychology, New Bulgarian University, Sofia, Bulgaria; 12 MASS Team, London, UK

**Keywords:** Adolescents, Delphi, DMD, Duchenne muscular dystrophy, Transition, Life expectancy

## Abstract

**Purpose:**

An increasing number of patients with Duchenne muscular dystrophy (DMD) now have access to improved standard of care and disease modifying treatments, which improve the clinical course of DMD and extend life expectancy beyond 30 years of age. A key issue for adolescent DMD patients is the transition from paediatric- to adult-oriented healthcare. Adolescents and adults with DMD have unique but highly complex healthcare needs associated with long-term steroid use, orthopaedic, respiratory, cardiac, psychological, and gastrointestinal problems meaning that a comprehensive transition process is required. A sub-optimal transition into adult care can have disruptive and deleterious consequences for a patient’s long-term care. This paper details the results of a consensus amongst clinicians on transitioning adolescent DMD patients from paediatric to adult neurologists that can act as a guide to best practice to ensure patients have continuous comprehensive care at every stage of their journey.

**Methods:**

The consensus was derived using the Delphi methodology. Fifty-three statements were developed by a Steering Group (the authors of this paper) covering seven topics: Define the goals of transition, Preparing the patient, carers/parents and the adult centre, The transition process at the paediatric centre, The multidisciplinary transition summary – Principles, The multidisciplinary transition summary – Content, First visit in the adult centre, Evaluation of transition. The statements were shared with paediatric and adult neurologists across Central Eastern Europe (CEE) as a survey requesting their level of agreement with each statement.

**Results:**

Data from 60 responders (54 full responses and six partial responses) were included in the data set analysis. A consensus was agreed across 100% of the statements.

**Conclusions:**

It is hoped that the findings of this survey which sets out agreed best practice statements, and the transfer template documents developed, will be widely used and so facilitate an effective transition from paediatric to adult care for adolescents with DMD.

**Supplementary Information:**

The online version contains supplementary material available at 10.1186/s13023-024-03270-2.

## Introduction

Duchenne muscular dystrophy (DMD) is a genetic disease that causes muscle weakness and wasting. Children born with DMD have a mutation on the dystrophin gene [1]. The dystrophin gene is located on the X chromosome; hence why DMD mostly affects boys [2]. However, female carriers may also rarely be affected and have muscle weakness and/or cardiomyopathy [3].

The dystrophin gene is composed of 79 exons coding for a protein of 3,685 amino acid residues [4]. It is a cohesive protein, linking actin filaments to other support proteins that reside on the inside surface of each muscle fibres’ plasma membrane (sarcolemma) [1, 2]. Absence of dystrophin, makes muscle fiber membranes fragile and susceptible to mechanical damage, results in progressive muscle degeneration, weakness and loss of function. This leads to loss of independent ambulation by the age of approximately 13 years, followed by a progressive cardiomyopathy and respiratory dysfunction [1, 2].

The standard of care of DMD aims to improve quality of life, delay disease progression and increase life expectancy; it requires a comprehensive multidisciplinary approach. Despite major therapeutic advances over the past 30 years, there is no cure for DMD [5]. Corticosteroids (glucocorticoids) aim to delay progression of the disease by reducing inflammation-induced muscle damage and thus loss of muscle strength and disease progression [6]. The improvement in supportive care and the extensive and early use of corticosteroids can increase life expectancy across into adulthood [7]. Life expectancy can be further lengthened by the increasing availability and use of disease modifying treatments [8–11].

The potential increase in life expectancy increases the requirement for an effective transition from paediatric to adult health services for adolescents with DMD, this being a crucial time for their future care [12, 13]. At the time when they have a desire for greater independence, adolescents and young adults with DMD often have increasing healthcare needs and physical reliance on others for activities of daily living. This can make a successful transition to adult lifestyles challenging [12, 13].

Due to the health problems that increase with age, a comprehensive multidisciplinary team (MDT) is needed to manage DMD patients, such as a paediatrician, general practitioner, pulmonologist, cardiologist, physical and rehabilitation specialist, orthopaedist, nutritionist, psychologist, and speech therapist [13].

There are concerns that a suboptimal transition may lead to a reduction in care quality and increases in emergency care. A systematic review assessed the impact on care after transition of paediatric patients with life-limiting conditions, such as DMD [14]. This review highlighted the possible numerous risks to care when a suboptimal transition occurs. These risks might include a reduction in adequate follow-up and therefore a reduction in the level of outpatient and supportive care, such as physiotherapy. Other consequences of suboptimal transition can include increases in inpatient admissions, inpatient bed days, emergency department visits and the requirement for supportive pharmaceutical support. As a consequence, quality of life might be negatively impacted and costs for providing care can increase significantly [14].

There is therefore an important need to make the transition of patients with DMD from paediatric to adult care optimal. To encourage that, healthcare teams require awareness and guidance on the preparation of the transition plan for their patients with DMD. To offer some guidance on a best practice transition process, we set up a Steering Group of paediatric and adult neurologists with experience of transitioning DMD patients to assist in the development and delivery of an activity that we hope will have a positive impact on the transition process moving forwards.

The objectives of this activity were to gain consensus, following the Delphi methodology, on the optimum approach to transitioning DMD patients from paediatric to adult care, covering several aspects of the ideal care pathway. Then, to develop a practical guide to support and guide physicians when transitioning DMD patients from paediatric to adult care to facilitate best practice for transition.

## Materials & methods

The sponsoring healthcare company (PTC Therapeutics) initiated and financially supported this consensus project, and commissioned The MASS Team, a healthcare consultancy, to independently facilitate and run this project in line with the Delphi methodology.

The initial Delphi phase was an exploration phase to identify the broad issues related to various components of the topic. Responses from this phase were edited and used to construct questionnaires for subsequent evaluation phase(s). Evaluation rounds were more specific, with questions seeking to rate or rank items in terms of their significance; and were analysed quantitatively. A valid Delphi methodology may consist of a number of rounds of voting on statements, depending on the level of agreement between respondents. There is anonymity of responses to remove undue social pressures from the process.

For this survey, the exploration phase took the form of a face-to-face meeting with ten experts (the Steering Group) in the management of patients with DMD from across Central Eastern Europe (CEE) to develop and refine statements about the transition process from paediatric to adult care. They developed 53 draft statements (see Appendix 1) that would be used in the subsequent evaluation phase.

Physicians for the exploration phase were suggested by the project sponsor, as the subject of this Delphi was not product specific this was not considered to be a conflict of interest. Neurologists with experience in DMD, treating paediatrics and adults were sought, with the aim of a 50:50 split. Members in the Steering Group are shown in Table 1.

**Table 1:** Steering Group members

Paediatric Neurologists	Adult Neurologists
Talya Dor (Israel)	Ana Maria Cobzaru (Romania)
Lenka Juříková (Czech Republic)	Amir Dori (Israel)
Ivan Litvinenko (Bulgaria)	Papadimas Georgios (Greece)
Léna Szabó (Hungary)	Maria Judit Molnar (Hungary)
Oana Aurelia Vladacenco (Romania)	Ivailo Tournev (Bulgaria)

The Steering Group defined the threshold for consensus in the evaluation phase as > 66%, with consensus being defined ‘high’ at > 66% and ‘very high’ at > 90% of respondents selecting ‘agree’ or ‘strongly agree’.

The evaluation phase was completed by 60 neurologists from six countries (Bulgaria, Czech Republic, Greece, Hungary, Israel, Romania). These countries were chosen for the research because the project sponsor, PTC Therapeutics international, wanted to support a study that examined treatment in the CEE region, as many studies are already focussed on western European countries.

Respondents were recruited by the Steering Group via a standardised email invitation. The evaluation phase survey was open from 27 February 2023 to 13 March 2023. Sixty eight percent of respondents were paediatricians. This phase consisted of 53 statements developed by the Steering Group during the exploration phase (see Appendix 1) that respondents were asked to agree or not agree with via a four-point Likert scale. It included questions about the transition of adolescent patients with DMD to adult care covering seven core themes:

- Define the goals of transition
- Preparing the patient, carers/parents and the adult centre
- The transition process at the paediatric centre
- The multidisciplinary transition summary – Principles
- The multidisciplinary transition summary – Content
- The first visit in the adult centre
- Evaluation of transition

A sample category and associated statements are shown in Table 2.

**Table 2:** Example of a category and associated statements for ranking for the level of agreement

Theme	Statement
Define the goals of transition	The goal of transition is to provide continuity of care to patients, as they move from paediatric to adult care
	The overall goal is to provide DMD patients with multidisciplinary co-ordinated care, regardless of the transition from paediatric to adult care
	The transfer process should be as positive an experience for the patient and parent(s) as possible
	Transition to adult care is mandatory, but must be safe and beneficial for the patients
	Enabling patients to take control of the management of their condition as an adult is one goal of transition

**Fig. 1:**
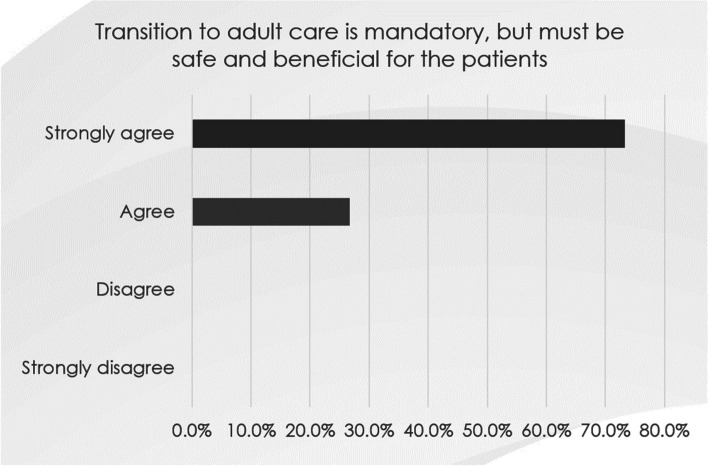
Example of respondents ranking a statement

Respondents were asked to rank each individual statement, ranging from ‘strongly disagree’ to ‘strongly agree’ [see Fig. 1 as an example]; they could also share specific comments. The results of the rankings were collated for each statement as per Table 3.

**Table 3:** Example of collated rankings for a sample of statements

Theme	Statement	Responders (n)	Overall Agreement	Consensus
Define the goals of transition	The goal of transition is to provide continuity of care to the patients, as they move from paediatric to adult care	60	98%	‘Very high’
	The overall goal is to provide DMD patients with multi-disciplinary co-ordinated care, regardless of the transition from paediatric to adult care	60	100%	‘Very high’
	The transfer process should be as positive an experience for the patient and parent(s) as possible	60	100%	‘Very high’
	Transition to adult care is mandatory, but must be safe and beneficial for the patients	60	100%	‘Very high’
	Enabling patients to take control of the management of their condition as an adult as one goal of transition	60	95%	‘Very high’

The results were collated and reviewed by the Steering Group, and are detailed below.

## Results

Data from 60 responders (54 full responses and six partial responses) were included in the final data set analysis. A consensus was agreed across 100% of the statements.

### Pre-Transition

#### Define the goals of transition

There was a ‘very high’ consensus for all statements to define the goals of transition developed by the Steering Group as presented in Table 4.

**Table 4:** Consensus results for statements defining the goals of transition

Theme	Statement	Responders (n)	Overall Agreement	Consensus
**Define the goals of transition**	The goal of transition is to provide continuity of care to patients, as they move from paediatric to adult care	60	98%	‘Very high’
	The overall goal is to provide DMD patients with multidisciplinary co-ordinated care, regardless of the transition from paediatric to adult care	60	100%	‘Very high’
	The transfer process should be as positive an experience for the patient and carers/parent(s) as possible	60	100%	‘Very high’
	Transition to adult care is mandatory, but must be safe and beneficial for the patients	60	100%	‘Very high’
	Enabling patients to take control of the management of their condition as an adult is one goal of transition	60	95%	‘Very high’

A 100% consensus was reached for statements confirming that the transition to adult care was mandatory but should be as positive for the patient and carers/parents as possible. It should include multidisciplinary co-ordinated care. The statement ‘The goal of transition is to provide continuity of care to patients, as they move from paediatric to adult care’ gained a 98% consensus. A ‘very high’ consensus of 95% was achieved for the statement ‘Enabling patients to take control of the management of their condition as an adult is one goal of transition’.

#### Preparing the patient, carers/parents and the adult centre

A ‘very high’ consensus was recorded for all statements relating to the preparation of the patient, carer/parent and adult centre for the transition, as presented in Table 5.

**Table 5:** Consensus results for statements on preparing the patients, carers/parents and adult centre for the transition

Theme	Statement	Responders (n)	Overall Agreement	Consensus
**Prepare patient/ parents + adult centre (written transition plan)**	Increasing patients’ knowledge of DMD from age 11 years will help them to better cope with the transition	59	97%	‘Very high’
	The need and the timeline for transition should be introduced to the patient and their parent(s) from age 14 years, if the patient is mature enough	59	93%	‘Very high’
	It is important patients and parents(s) accept the need to transfer	59	100%	‘Very high’
	The Paediatric Neurologist should identify potential adult centres for the patient to transfer to	59	98%	‘Very high’
	The Paediatric Neurologist is responsible for drafting the transfer plan	59	95%	‘Very high’
	Patient and parent(s) expectations of transfer should be discussed with the Paediatric Neurologist	59	98%	‘Very high’
	The Paediatric Neurologist should ensure the patient’s and parent(s) expectations are realistic	59	100%	‘Very high’
	The Paediatric Neurologist refines the transfer plan with input from the patient and parent(s)	59	100%	‘Very high’

There was a 100% consensus on the statements ‘It is important patients and parents(s) accept the need to transfer’, ‘The Paediatric Neurologist should ensure the patient’s and parent(s) expectations are realistic’, and ‘The Paediatric Neurologist should refine the transfer plan with input from the patient and parent(s)’.

The need to include the patient and carers/parents in the development of the transfer plan had a 98% consensus for the statement ‘Patient and parent(s) expectations of transfer should be discussed with the Paediatric Neurologist’ and there was a 97% consensus for the statement ‘Increasing patients’ knowledge of DMD from age 11 will help them better cope with the transition’.

The statement ‘The Paediatric Neurologist should identify potential adult centres for the patient to transfer to’ gained a 98% consensus and the statement ‘The Paediatric Neurologist is responsible for drafting the transfer plan’ had a 95% consensus.

The statement ‘The need and the timeline for transition should be introduced to the patient and their parent(s) from age 14, if the patient is mature enough’ achieved a 93% consensus.

### The Transition

#### The transition process at the paediatric centre

The actual transition process at the paediatric centre will have a significant impact on the success of transition from paediatric to adult care. The statements in the survey focussed on communications between paediatric and adult neurologists, and between healthcare professionals and patients/parents/carers.

There was a ‘very high’ consensus for all statements relating to transition processes at the paediatric centre, as shown in Table 6. A 100% consensus for the statements ‘It is important for all stakeholders to accept that there may be a need for flexibility in the transfer process’ and ‘Following the joint meeting with the patient and parent(s), the Paediatric and Adult Neurologists should communicate to align on the patient’s needs and expectations’ was recorded. The statement ‘The Paediatric Neurologist is responsible for ensuring the patient and parent(s) are prepared for the first meeting with the Adult Neurologist’ had a 95% consensus and the statement ‘It is important that the Paediatric and Adult Neurologists have a joint meeting (either face to face or virtual) with the patient and parent(s) in an environment the patient is familiar with’ gained a 91% consensus.

**Table 6:** Consensus results for statements on the transition process at the paediatric centre

Theme	Statement	Responders (n)	Overall Agreement	Consensus
**Transition process at the paediatric centre**	The Paediatric Neurologist is responsible for ensuring the patient and parent(s) are prepared for the first meeting with the Adult Neurologist	58	95%	‘Very high’
	It is important that the Paediatric and Adult Neurologists have a joint meeting (either face to face or virtual) with the patient and parent(s) in an environment the patient is familiar with	58	91%	‘Very high’
	It is important for all stakeholders to accept that there may be a need for flexibility in the transfer process	58	100%	‘Very high’
	Following the joint meeting with the patient and parent(s), the Paediatric and Adult Neurologists should communicate to align on the patient’s needs and expectations	58	100%	‘Very high’

#### The multidisciplinary transition summary—principles

The multidisciplinary transition summary should be a comprehensive document of clinical and non-clinical details of the adolescent patient with DMD to facilitate a smooth transition process—and ultimately a smooth transfer—into adult care. A template document for the Multidisciplinary Transition Summary can be found in Appendix 2.

There was a ‘high’ or ‘very high’ consensus to statements on the principles of the Multidisciplinary Transition Summary, as detailed in Table 8. There was a 100% consensus for the statement ‘The most up to date information should be included in the Multidisciplinary Transition Summary’. Ninety eight percent consensus was recorded for the two statements ‘For consistency, there should be a standard format for the Multidisciplinary Transition Summary’ and ‘The Multidisciplinary Transition Summary should include medical and non-medical information in separate sections’.

A ‘high’ consensus of 89% was recorded for the statements ‘Preparation of the Multidisciplinary Transition Summary should be the responsibility of the Paediatric Neurologist’ and ‘The Multidisciplinary Transition Summary should be completed just prior to transfer to the adult centre’ as detailed in Table 7.

**Table 7:** Consensus results for statements on the Multidisciplinary Transition Summary principles document

Theme	Statement	Responders (n)	Overall Agreement	Consensus
**Multidisciplinary Transition Summary—Principles**	Preparation of the Multidisciplinary Transition Summary should be the responsibility of the Paediatric Neurologist	57	89%	‘High’
	The Multidisciplinary Transition Summary should be completed just prior to transfer to the adult centre	57	89%	‘High’
	For consistency, there should be a standard format for the Multidisciplinary Transition Summary	57	98%	‘Very high’
	The most up to date information should be included in the Multidisciplinary Transition Summary	57	100%	‘Very high’
	The Multidisciplinary Transition Summary should include medical and non-medical information in separate sections	57	98%	‘Very high’

#### The multidisciplinary transition summary – content

As previously stated, the Multidisciplinary Transition Summary should be a very comprehensive document of clinical and non-clinical details on the adolescent patient with DMD. The Steering Group developed a number of statements (n = 14) for the survey that covered multiple features of a comprehensive transition.

The consensus endorsed opinions of the Steering Group that the Multidisciplinary Transition Summary should include numerous clinical and non-clinical details of the patient so that Paediatric Neurologists can share as much information on the patient as possible with Adult Neurologists. Those patient details include the patient’s age, clinical parameters and test results, family details, cardiac and pulmonary status.

A ‘very high’ consensus was achieved for all statements regarding the content of the Multidisciplinary Transition Summary, as detailed in Table 8. One hundred percent consensus was achieved for eight out of fourteen statements.

**Table 8:** Consensus results for statements on the Multidisciplinary Transition Summary content

Theme	Statement	Responders (n)	Overall Agreement	Consensus
**Multidisciplinary Transition Summary—Content**	The patient’s age should be included in the Multidisciplinary Transition Summary	56	98%	‘Very high’
	Details of family, such as siblings and socio-economic status, should be included in the Multidisciplinary Transition Summary	56	98%	‘Very high’
	Details of genetic mutations should be included in the Multidisciplinary Transition Summary	56	100%	‘Very high’
	Cardiac status details, including latest test results and name and contact details of the cardiologist, should be included in the Multidisciplinary Transition Summary	56	100%	‘Very high’
	Pulmonary status details, including latest test results and name and contact details of the specialist, should be included in the Multidisciplinary Transition Summary	56	100%	‘Very high’
	Current details of the patient’s neuropsychological status, including level of cognition, emotional maturity and social independence, should be included in the Multidisciplinary Transition Summary	56	100%	‘Very high’
	Current details of the patient’s endocrinological status, including bone health, and puberty status, weight/obesity, diet, and metabolic issues, should be included in the Multidisciplinary Transition Summary	56	100%	‘Very high’
	Up to date functional status including ambulatory status (including how long they have been using aids, such as wheelchairs), upper limb function, 6MWT & other relevant test results, potential for independent living and occupational therapy support required (and OT assessments if available) QoL measures and driving licence status should be included in the Multidisciplinary Transition Summary	56	100%	‘Very high’
	The most up to date treatment information (pharmacological and other), treatment history and details of any involvement in clinical trials should be included in the Multidisciplinary Transition Summary	56	100%	‘Very high’
	Details of previous hospitalisations and operations (in particular orthopaedic), should be included in the Multidisciplinary Transition Summary	56	98%	‘Very high’
	Information on comorbid conditions should be included in the Multidisciplinary Transition Summary	56	100%	‘Very high’
	Input from the social worker should be included in the Multidisciplinary Transition Summary	56	93%	‘Very high’
	The most recent gastroenterology history should be included in the Multidisciplinary Transition Summary	56	95%	‘Very high’
	Vaccination history should be included in the Multidisciplinary Transition Summary	56	98%	‘Very high’

### The first visit in an adult centre post-transition

#### The first visit in the adult centre

Results of this survey indicated that there was a ‘very high’ consensus for all statements regarding the patients’ first visit in the adult centre, as shown in Table 9. The responders agreed that, at the first visit, the Adult Neurologist should:

- Refamiliarise themselves with the patients Multidisciplinary Transition Summary and perform an examination of the patient
- Identify the most appropriate co-ordinator who should hold the information and recommendations from all specialties, to reduce the burden on the patient and the Adult Neurologist
- Discuss expectations of treatment and life aspirations with the patient and parent(s) and, if possible, spend some time discussing these topics alone with the patient
- Check the patients’ level of knowledge of DMD and adherence to treatment
- Provide the patient with a main point of contact at the adult centre
- Establish the patient’s preference for being seen with caregiver or alone
- Advise the patient that they will be the person who will be responsible for providing necessary data to any registries
- Create a plan for how they will approach the patient’s care
- Organise consultations with other specialties as required

**Table 9:** Consensus results for statements on the first visit in the adult centre

Theme	Statement	Responders (n)	Overall Agreement	Consensus
**First visit in adult centre**	At the first visit, the Adult Neurologist should refamiliarise themselves with the patients Multidisciplinary Transition Summary and then perform an examination of the patient	54	100%	Very high consensus
	At the first visit, the Adult Neurologist should discuss expectations of treatment and life aspirations with the patient and parent(s) and if possible, spend some time discussing these topics alone with the patient	54	98%	Very high consensus
	At the first visit, the Adult Neurologist should establish the patient’s preference for being seen with caregiver or alone	54	93%	Very high consensus
	At the first visit, the Adult Neurologist should check the patients’ level of knowledge of DMD and adherence to treatment	54	100%	Very high consensus
	At the first visit, the Adult Neurologist should provide the patient with a main point of contact at the adult centre	54	100%	Very high consensus
	At the first visit, the Adult Neurologist should advise the patient that they will be the person who will be responsible for providing necessary data to any registries	54	96%	Very high consensus
	At the first visit, the Adult Neurologist should identify the most appropriate co-ordinator who should hold the information and recommendations from all specialties, to reduce the burden on the patient and the Adult Neurologist	54	100%	Very high consensus
	At first visit, the Adult Neurologist (or case manager if available) should organise consultations with other specialties as required	54	94%	Very high consensus
	Each member of the multidisciplinary team should individualise the frequency of appointments to suit the patient’s specific needs	54	96%	Very high consensus
	After the first visit, the Adult Neurologist should create a plan for how they will approach the patient’s care	54	100%	Very high consensus
	The follow-up to the first visit should be individualised according to patient needs and preferences (including frequency of visits and assessments)	54	98%	Very high consensus

There was also a ‘very high’ consensus that the follow-up to the first visit should be individualised according to patient needs and preferences (including frequency of visits and assessments), and that each member of the MDT should individualise the frequency of appointments to suit the patient’s specific needs.

### Post-transition

#### Evaluation of the transition

A ‘very high’ consensus was achieved for five of the six statements. Those statements supported the opinion that the Adult Neurologist should co-ordinate and collate feedback for the post-transition evaluation, discuss the feedback with other members of the core care team to identify any issues and solutions and that, when they are satisfied, they can confirm the transition is complete. The sixth statement achieved a ‘high’ consensus of 78% and proposed that the post-transition evaluation should be conducted approximately six months after the transition to adult care. See details in Table 10.

**Table 10:** Consensus results for statements on the evaluation of the transition

Theme	Statement	Responders (n)	Overall Agreement	Consensus
**Evaluation of the transition**	Approximately six months after transition to adult care, an evaluation should be conducted with the patient, parent(s), paediatric and adult care teams to evaluate the process	54	78%	‘High’
	The Adult Neurologist should co-ordinate the collation of feedback for the post-transition evaluation	54	94%	‘Very high’
	Post-transition evaluation should enable comparison between respondents, with some questions specifically to highlight any differences between patients/parent(s) and the medical teams	54	96%	‘Very high’
	The questions for the post-transition evaluation should be specific, focussed on the transition and include some open-ended questions; questions should not be subjective	54	93%	‘Very high’
	As part of the post-transition evaluation, the Adult Neurologist should discuss the feedback with other members of the core team and identify any issues and solutions	54	98%	‘Very high’
	When post-transition feedback is collected and the Adult Neurologist is satisfied, the transition can be confirmed as complete	54	96%	‘Very high’

### Transfer plan

**Fig. 2:**
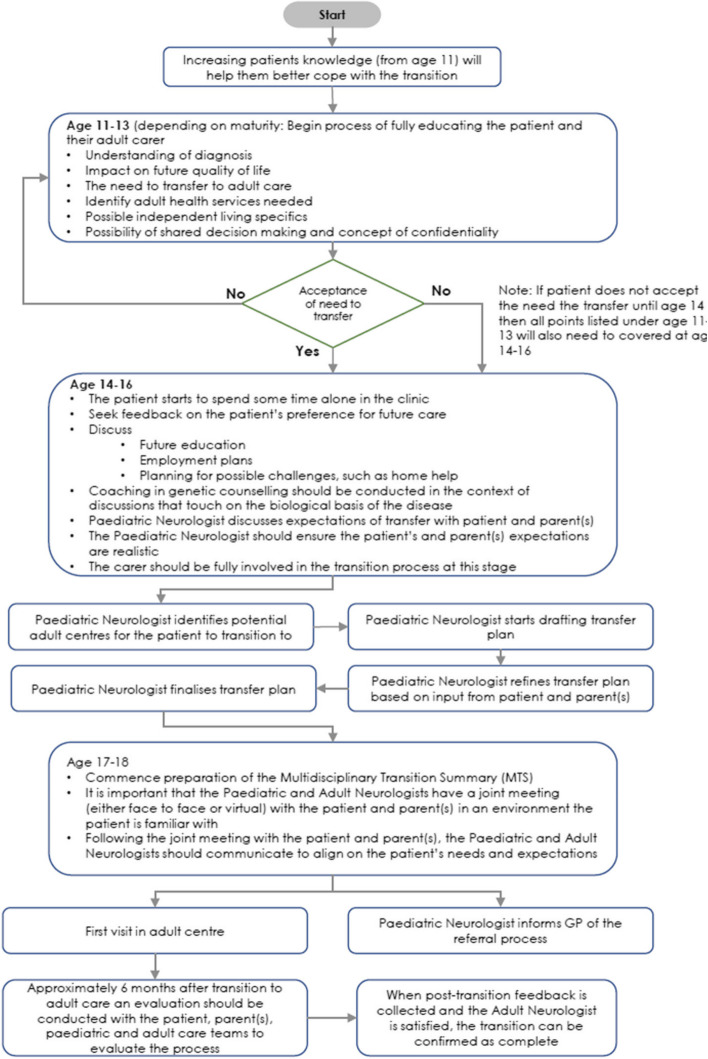
Sample template for Transfer Plan. Adapted from Molnár MJ [15]

The survey results indicated the important need to make the transition of patients with DMD from paediatric to adult care optimal and, to encourage that, healthcare teams require awareness and guidance on the preparation of the transition plan for their patients with DMD. The survey results also indicated key elements of that process for the preparation of a transition plan. The Steering Group therefore collaborated to develop a template for an effective Transfer Plan (Appendix 2) to aid Paediatric Neurologists understanding of the necessary planning and steps towards an optimal transition of paediatric DMD patients into adult care. The plan has been created as a flow diagram [Fig. 2] illustrating the possible steps of the transition which Paediatric Neurologists can easily follow.

### Transition summary document

The results of the survey indicated that that, to encourage best practice to generate transfer documentation for the transition of DMD patients to adult care, a template of a summary document for the paediatric neurologist would be beneficial. The Steering Group collaborated to develop a template for an effective Transition Summary Document (Appendix 3). The template Transition Summary Document is effectively a template ‘form’ covering the detail that a Paediatric Neurologist might aim to include in the Transition Summary Document for the Adult Neurologist and MDT. The template document includes sections for documentation, such as:

- Patient contact details
- Details of medical providers
- School or work details
- Genetics
- Medications
- Safety
- Disease education
- Involvement in clinical trials
- Medical history
- Current functional status
- Assessments and quality of life
- Equipment use

Based on the results of this survey, it is hoped that Paediatric Neurologists will find this template Transition Summary Document useful, and that it will be easier for them to develop the necessary documentation to facilitate an optimal transition into adult care.

## Discussion

This paper details the results of a survey of clinicians, following the Delphi methodology, to gain consensus on the optimum approach to transitioning DMD patients from paediatric to adult care.

The potential increase in life expectancy for patients with DMD increases the requirement for the effective transition from paediatric to adult health services. Just when they have a desire for greater independence, adolescents and young adults with DMD often have increasing healthcare needs and physical reliance on others for activities of daily living. This can make a successful transition to adult lifestyles challenging [12, 13].

The Steering Group felt it was important for paediatric and adult care teams to agree on the goals of transition for DMD patients from paediatric to adult care. If the goals are clear, the transition process itself can be implemented more optimally. A 100% consensus was reached for statements confirming that the transition to adult care was mandatory but should be as positive for the patient and carers/parents as possible, and should include the provision of multidisciplinary co-ordinated care. The need to include the patient and carers/parents in the development of the Transfer Plan achieved a 98% consensus.

It was also agreed that, when patients are prepared appropriately for their transition to adult care, transition would be more successful. The results of this survey indicated that responders acknowledged that the patient must understand the need for their transfer to adult care, and that their expectations are realistic; this should increase the likelihood that transition will be successful. It was also clear from the survey results that the process and timeline in different countries/at different centres will vary dependent on resourcing. There were some differences in results from responders with regards to preparing the patients and family for the transfer, with some slightly lower percentage consensus received for some statements from the Czech Republic and Israel; however, the Steering Group felt that this possibly was due to language differences.

In order to achieve an effective and positive transition from paediatric to adult care, it should be acknowledged that one part of the wider transition is from dependent child to independent adult. It is important that the transfer to adult care should not occur before the adolescent has the necessary skills and education to manage their illness largely independently of parents and staff —skills they are unlikely to be taught in the adult clinic. So, to achieve this, preparation must begin well before the anticipated transition time—preferably in early adolescence, when a series of educational interventions should discuss a patients’ understanding of their disease, the treatment rationale, their source of symptoms, how to recognise deterioration and take appropriate action and, most importantly, how to seek help from their healthcare team. Leaflets and materials about the transition programme, and details of the adult service, should be provided in clinic settings from early adolescence.

At first visit to the adult centre by the transitioning patient, the adult MDT must be prepared, communication with the patient should be clear and positive, and any treatments should be uninterrupted [13]. There was a ‘very high’ consensus for all statements regarding the patients’ first visit in the adult centre, with responders agreeing that, at the first visit, the Adult Neurologist should be proactive at checking the patients’ level of knowledge, create a care plan and provide the patient with details of their continued care, as well as involve the MDT and provide the patient with a point of contact.

There was also a ‘very high’ consensus that the follow-up to the first visit should be individualised according to patient needs and preferences (including frequency of visits and assessments), and that each member of the MDT should individualise the frequency of appointments to suit a patient’s specific needs.

A country breakdown of the survey results highlighted that there was no consensus from responders from Israel or the Czech Republic concerning if or when a post-transition evaluation should be conducted. The Steering Group discussed this and felt that the lack of consensus in Israel was due to the fact that it is not standard practice as, typically, the Paediatric Neurologist sends a letter to the Adult Neurologist. In the Czech Republic, it is not usually an issue for Adult and Paediatric Neurologists to meet face-to-face; it is possible that some responders felt that Adult Neurologists would feel it was not necessary to meet at that time.

The survey responders agreed that the Paediatric Neurologist should be responsible for drafting the Transfer Plan, indicating how important a role Paediatric Neurologists have in the transition process. They also agreed that the process and timeline of the transition will vary dependent on resourcing and the maturity of the patient. The survey also indicated that communication between physicians and patients/parents/carers and between Paediatric and Adults Neurologists are considered important to a successful transition process.

There was also a ‘very high’ consensus that the Multidisciplinary Transition Summary document should be in a standard format and that it should include up to date medical and non-medical information. Due to the health problems that increase with age, an array of specialists is necessary, such as paediatrician, general practitioner, pulmonologist, cardiologist, physical and rehabilitation specialist, orthopaedist, nutritionist, psychologist, and speech therapist [13].

A possible limitation of this study is the utilisation of the clinicians in the Steering Group to recruit respondents for the evaluation phase, as this may have introduced selection bias into the process. The number of respondents from Bulgaria (as reported in Table 11) represented 43% of all respondents, as such an analysis including and excluding Bulgaria was conducted to determine if this impacted the study results. However, results of the analysis of the data, with and without the inclusion of the responders from Bulgaria, indicated that in most cases, this majority had little impact on the level of consensus. The exception being a statement regarding completion of an evaluation phase six months post transfer, which failed to reach consensus when responders from Bulgaria were excluded from the analysis. (Table 12).

**Table 11:** Number of responders per country

Country	Number of responders
Bulgaria	26
Czech Republic	9
Greece	7
Hungary	10
Israel	4
Romania	4
**Total**	**60**

**Table 12:** Analysis of the data with and without the inclusion of the responders from Bulgaria

Theme	Statement	All countries	Excluding Bulgaria
Prepare patient/ parents + adult centre (written transition plan)	The need and the timeline for transition should be introduced to the patient and their parent(s) from age 14, if the patient is mature enough	Very high consensus	High consensus
Transition processes at Paediatric centre	It is important that the Paediatric and Adult Neurologists have a joint meeting (either face to face or virtual) with the patient and parent(s) in an environment the patient is familiar with	Very high consensus	High consensus
Multidisciplinary Transition Summary—Principles	Preparation of the Multidisciplinary Transition Summary should be the responsibility of the Paediatric Neurologist	High consensus	Very high consensus
	The Multidisciplinary Transition Summary should be completed just prior to transfer to the adult centre	High consensus	Very high consensus
Evaluation	Approximately 6 months after transition to adult care an evaluation should be conducted with the patient, parent(s), paediatric and adult care teams to evaluate the process	High consensus	No consensus
	The questions for the post-transition evaluation should be specific, focussed on the transition and include some open-ended questions; questions should not be subjective	Very high consensus	High consensus

Another possible limitation of this study is the focus on the overall transition process, without specific consideration of any differences in the healthcare systems and resources in the different countries. However, the aim of the Steering Group was to create a general transition plan whilst accepting that differences between healthcare systems might mean that some adjustment would be required to implement in different countries. At the centres of the Steering Group members, the status of a formal transition process varies. In Hungary, for instance, the processes proposed in this paper have been implemented whilst in Bulgaria, although there is no formal plan in place, the communication level between adult and paediatric neurologists is at a very high level during patient transition across to adult care, which is due in part to adult and paediatric patients being treated in the same hospital. A formal plan for transition in the other countries is yet to be formalised.

Using the consensus results, the Steering Group developed templates for a Multidisciplinary Transition Summary and Multidisciplinary Transition Plan; it is hoped that these will guide neurologists through the transition process in DMD and encourage best practice.

Many patients with DMD now have an increased life expectancy as a result of medical interventions. Consequently, there are an increasing number of adolescent DMD patients transitioning from paediatric- to adult-oriented healthcare [7–11].

Adolescents and adults with DMD have unique and highly complex healthcare needs associated with long-term steroid usage, cardiac, orthopaedic, respiratory, psychological and gastrointestinal problems. It is, therefore, essential that the transition from adolescent to adult clinics is comprehensive, providing uninterrupted care. With little official guidance on this, this can be a challenge.

This Delphi consensus provides this much-needed guidance on the nature and details of an effective transition for DMD patients. It is hoped that the findings of this survey, and the templates (Appendix 2 & 3) for a Multidisciplinary Transition Summary and Multidisciplinary Transition Plan developed by the Steering Group and presented here, will enable neurologists to transition adolescents with DMD from paediatric to adult care in an optimal and more standardised manner.

### Supplementary Information

Supplementary Material 1.

Supplementary Material 2.

Supplementary Material 3.

Supplementary Material 4.

## Data Availability

All data supporting the findings of this study are available within the paper and its Supplementary Information, including the raw and analysed data from the evaluation phase.

## Abbreviations

CEE: Central Eastern Europe
DMD: Duchenne muscular dystrophy
nmDMD: Nonsense mutation Duchenne muscular dystrophy
MDT: Multidisciplinary team
OT: Occupational therapy
QoL: Quality of life
