# Supplementary figures and images for: Essential components of an effective transition from paediatric to adult neurologist care for adolescents with Duchenne muscular dystrophy; a consensus derived using the Delphi methodology in Eastern Europe, Greece and Israel
Source: Orphanet J Rare Dis. 2024 Jul 9;19:260. doi: 10.1186/s13023-024-03270-2 (PMC11234532; doi:10.1186/s13023-024-03270-2)

**Appendix 1**

Survey questions


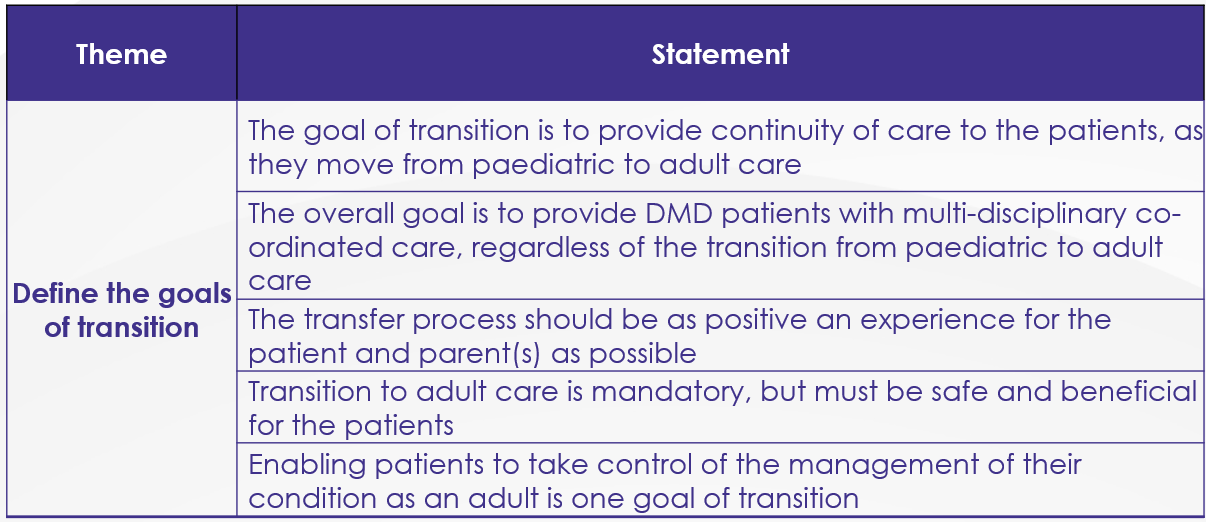


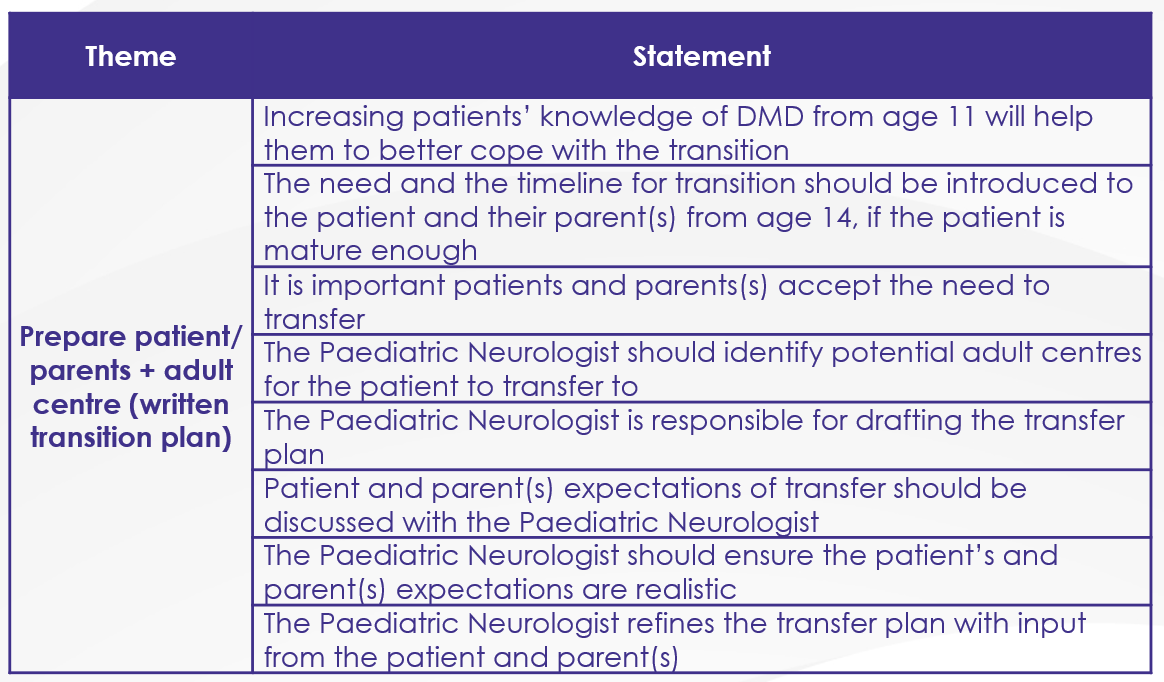


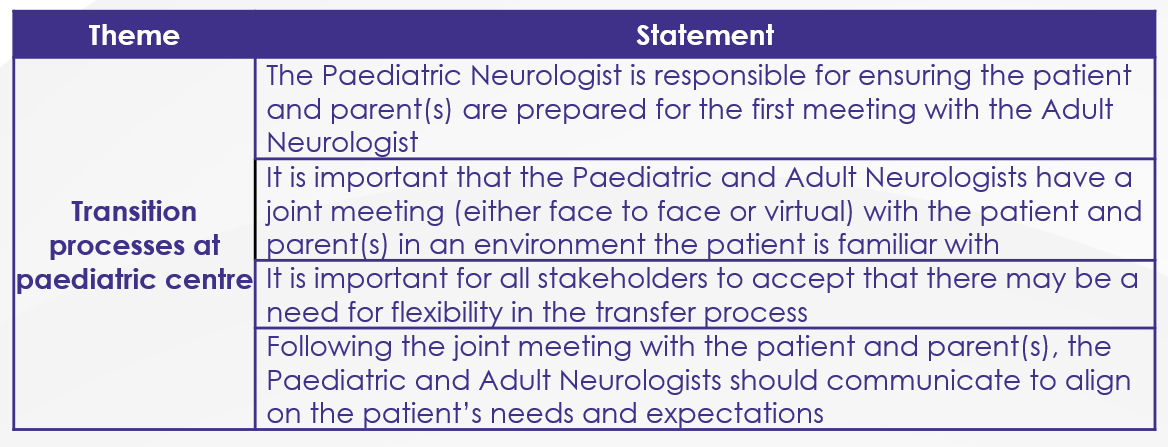


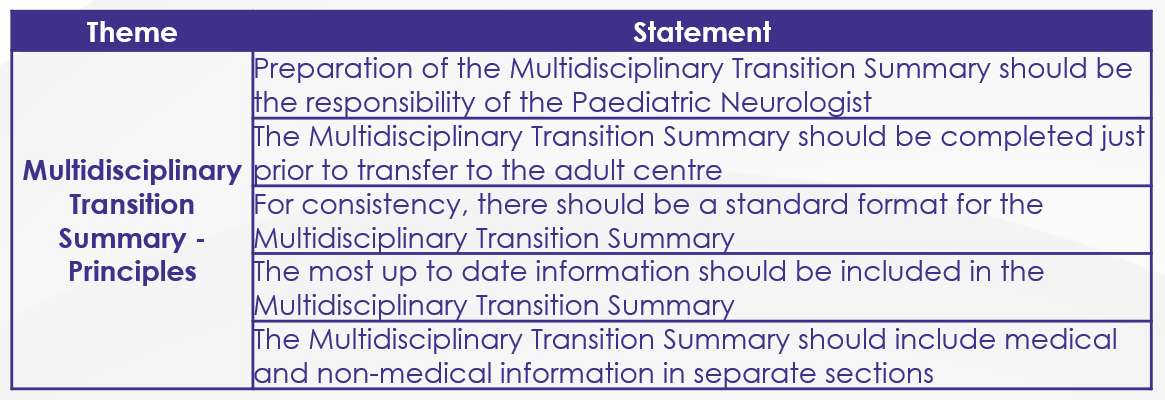


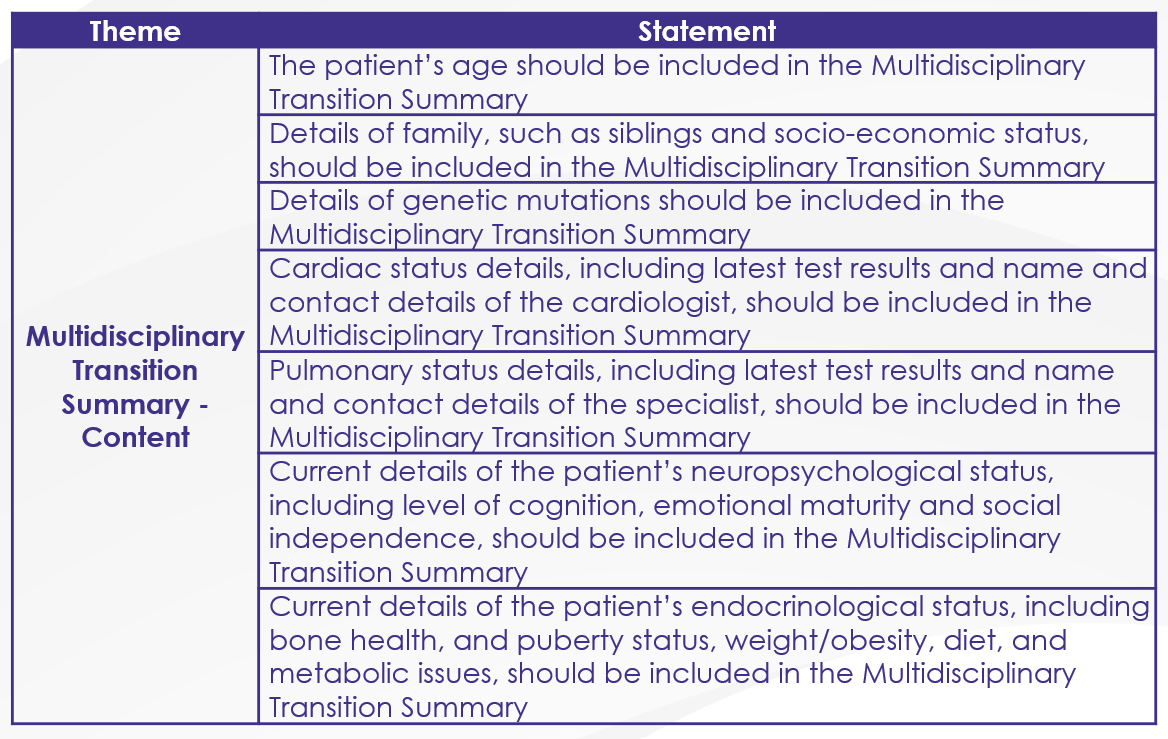


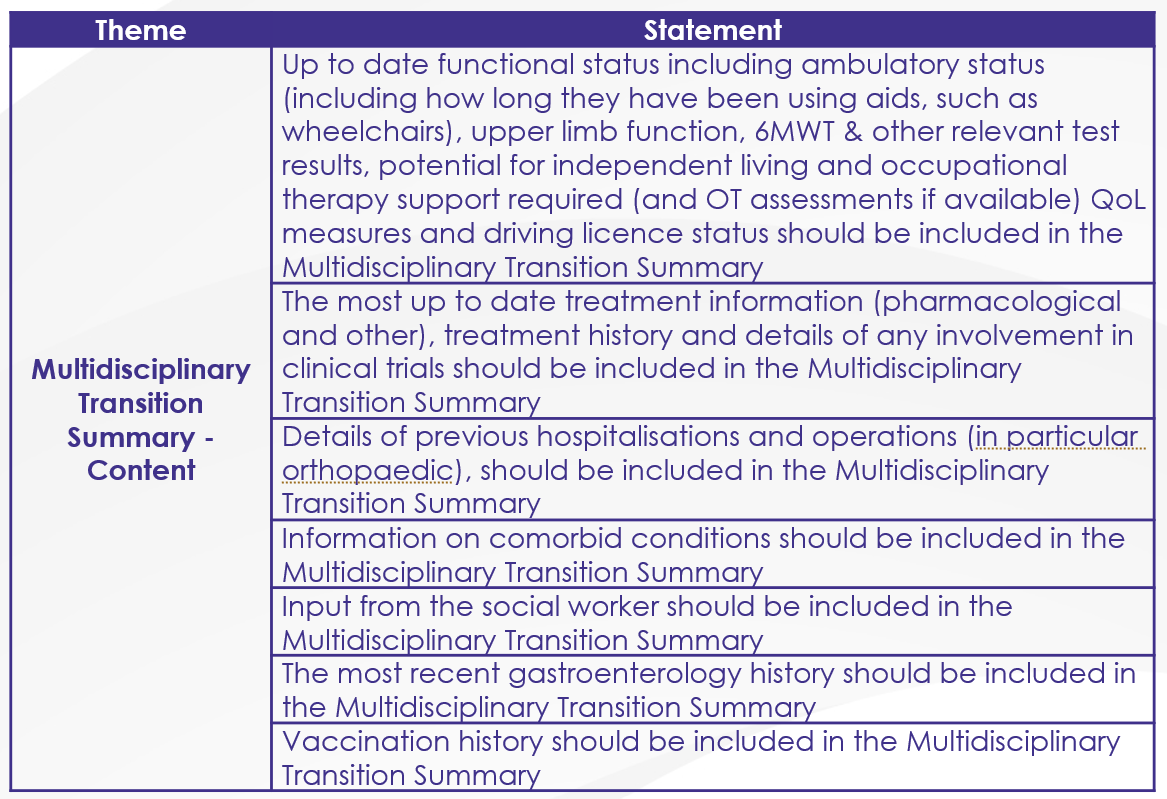


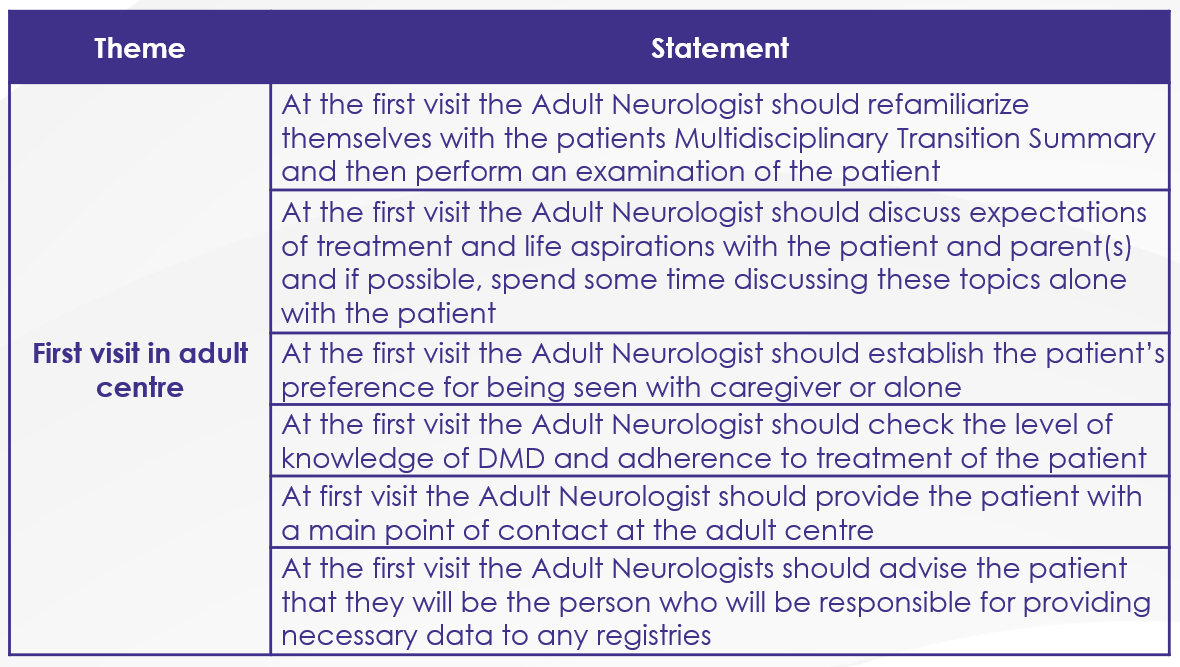


**
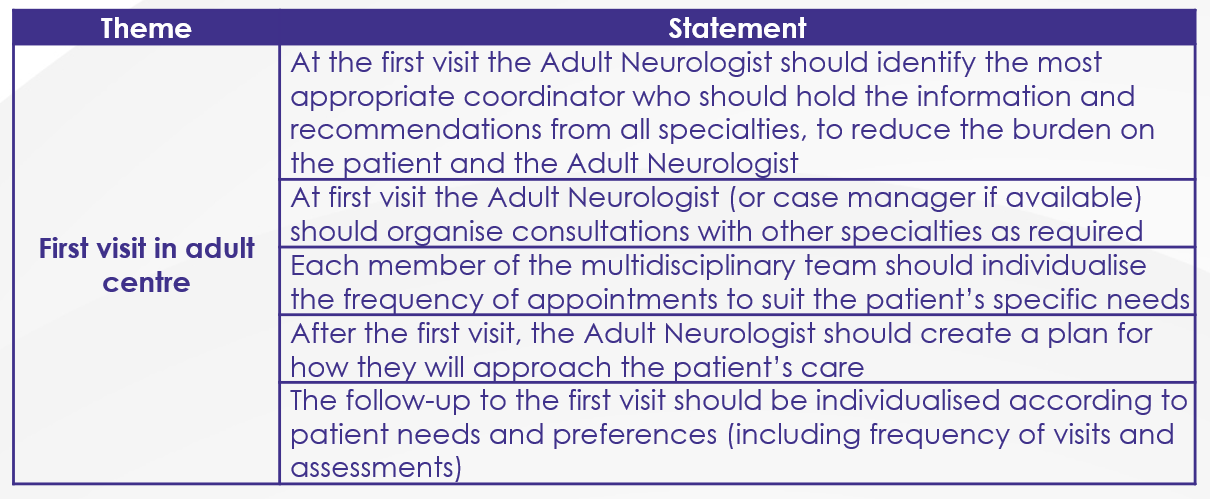
**

**
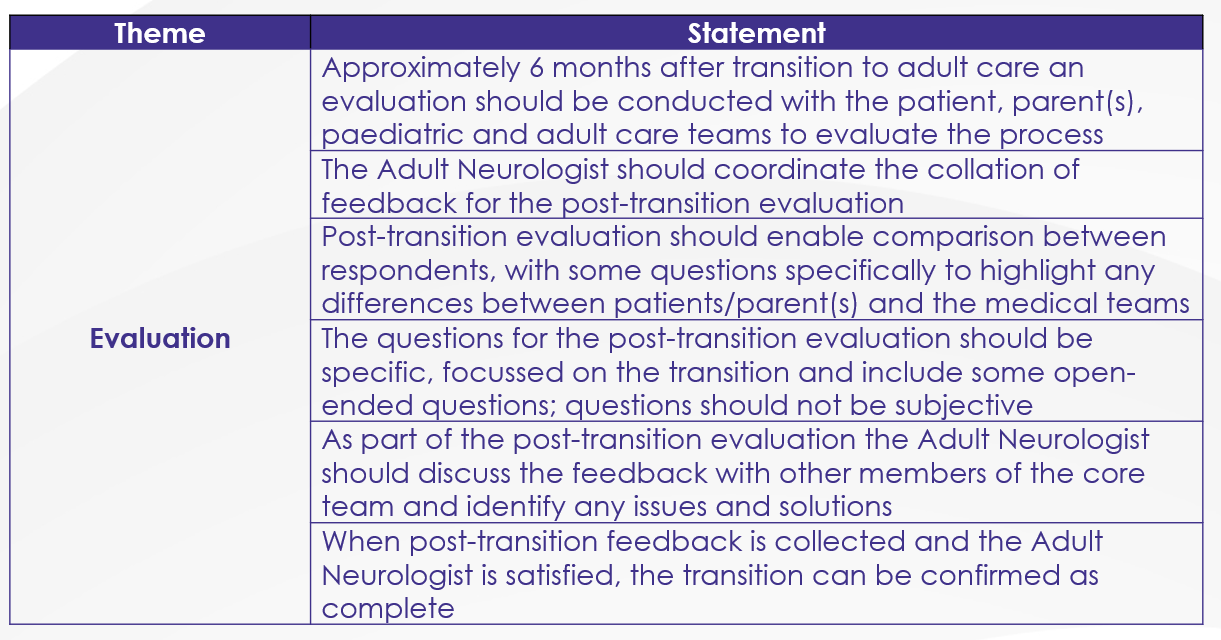
**

Supplement: Supplementary file 1 — Supplementary Material 1. [file 13023_2024_3270_MOESM1_ESM.docx]

**Appendix 2**

Multidisciplinary Transition Plan Template

**
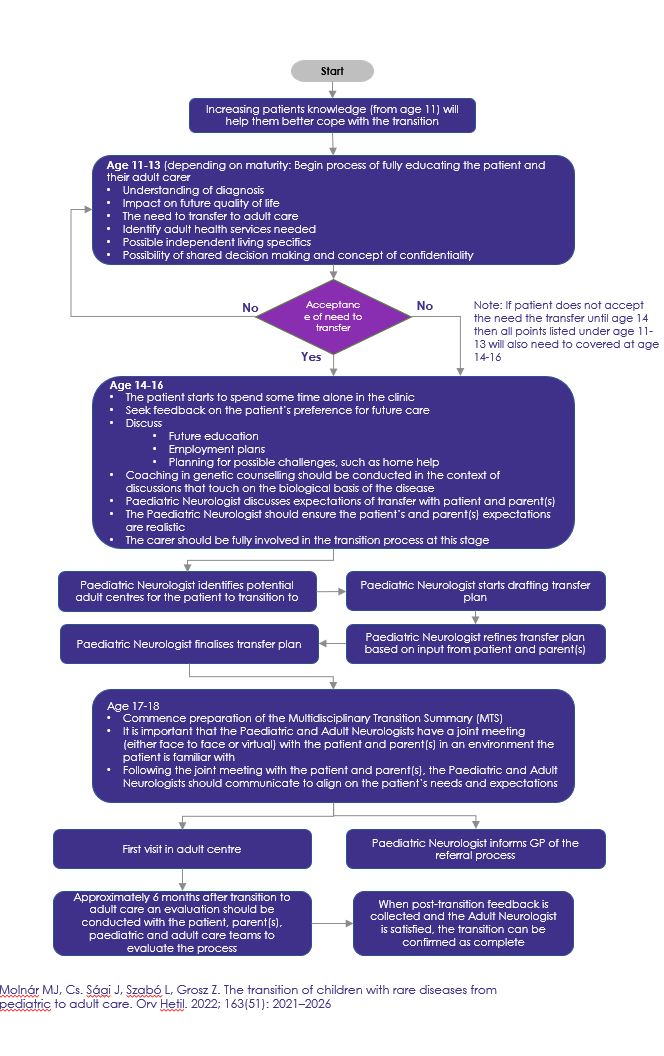
**

Supplement: Supplementary file 2 — Supplementary Material 2. [file 13023_2024_3270_MOESM2_ESM.docx]

**Appendix 3**

Multidisciplinary Transition Summary Template
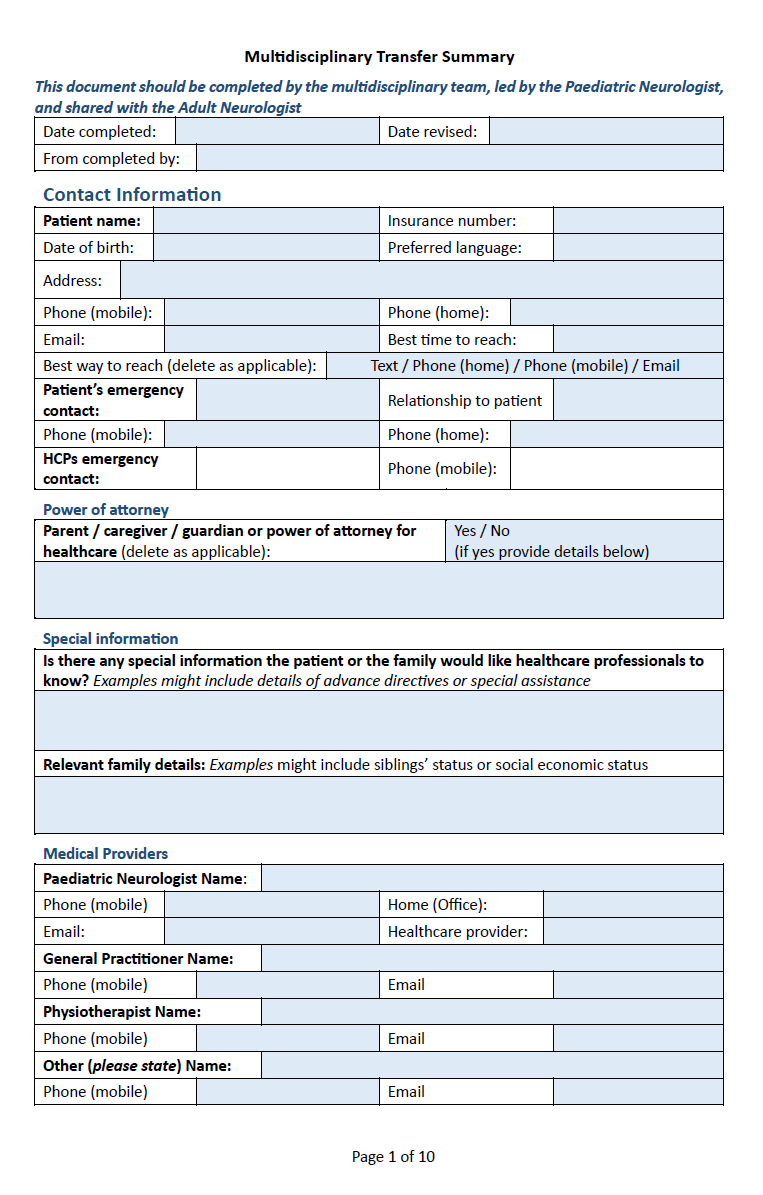


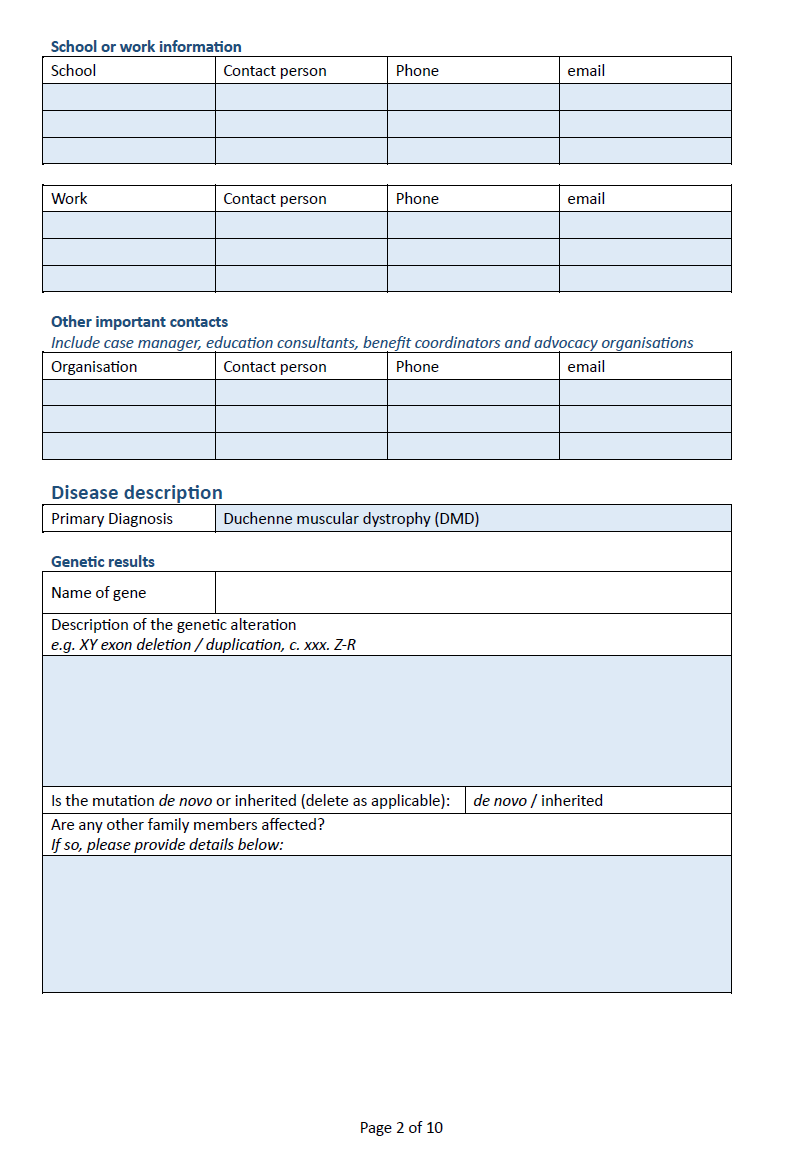


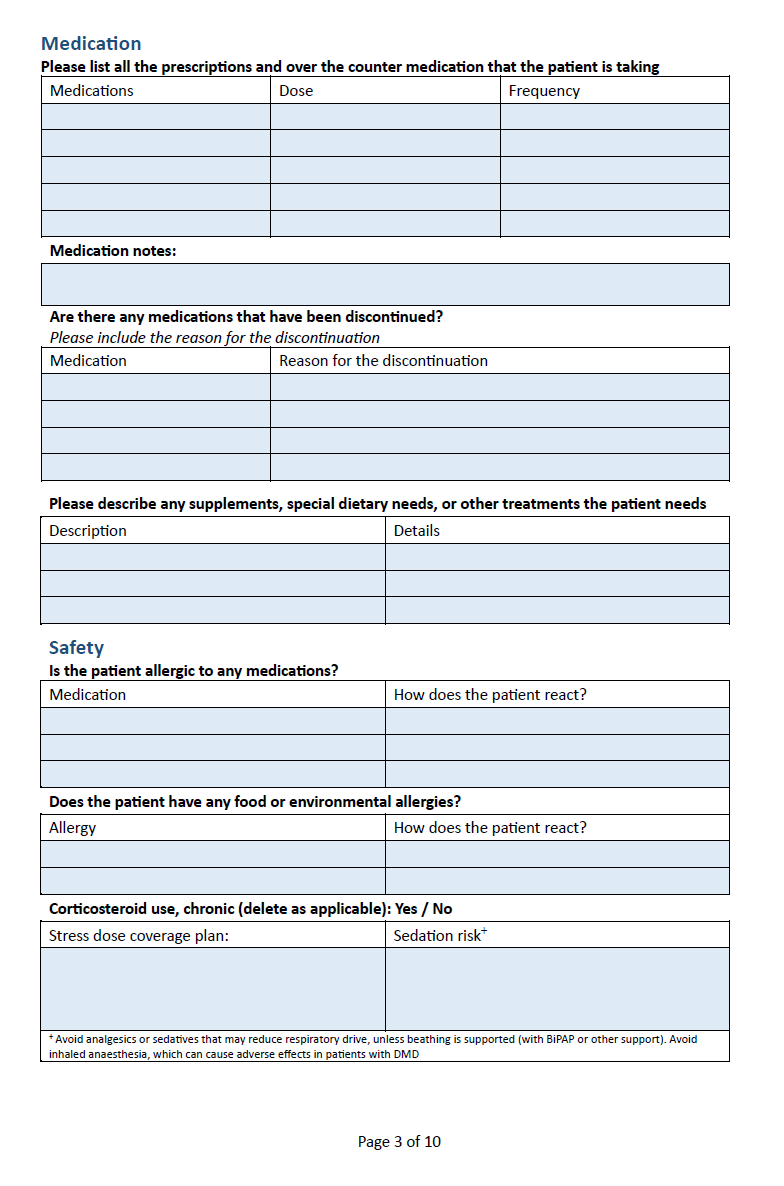


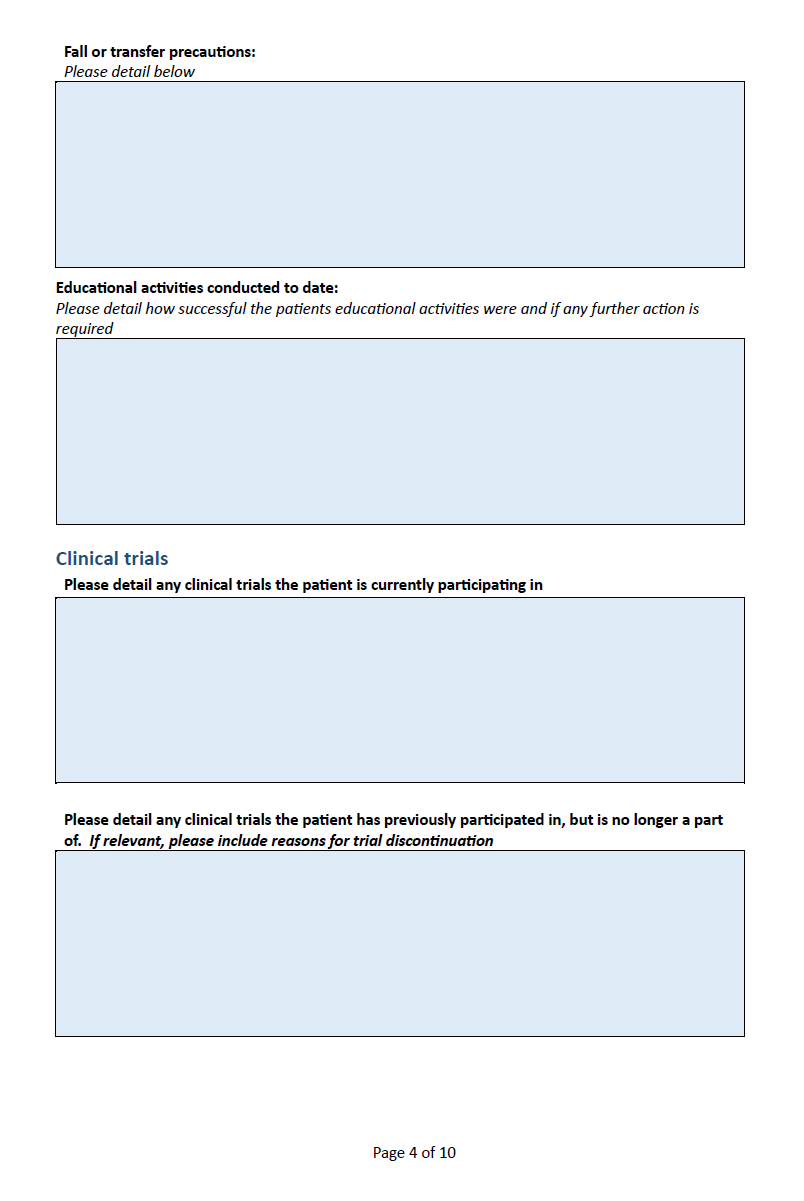


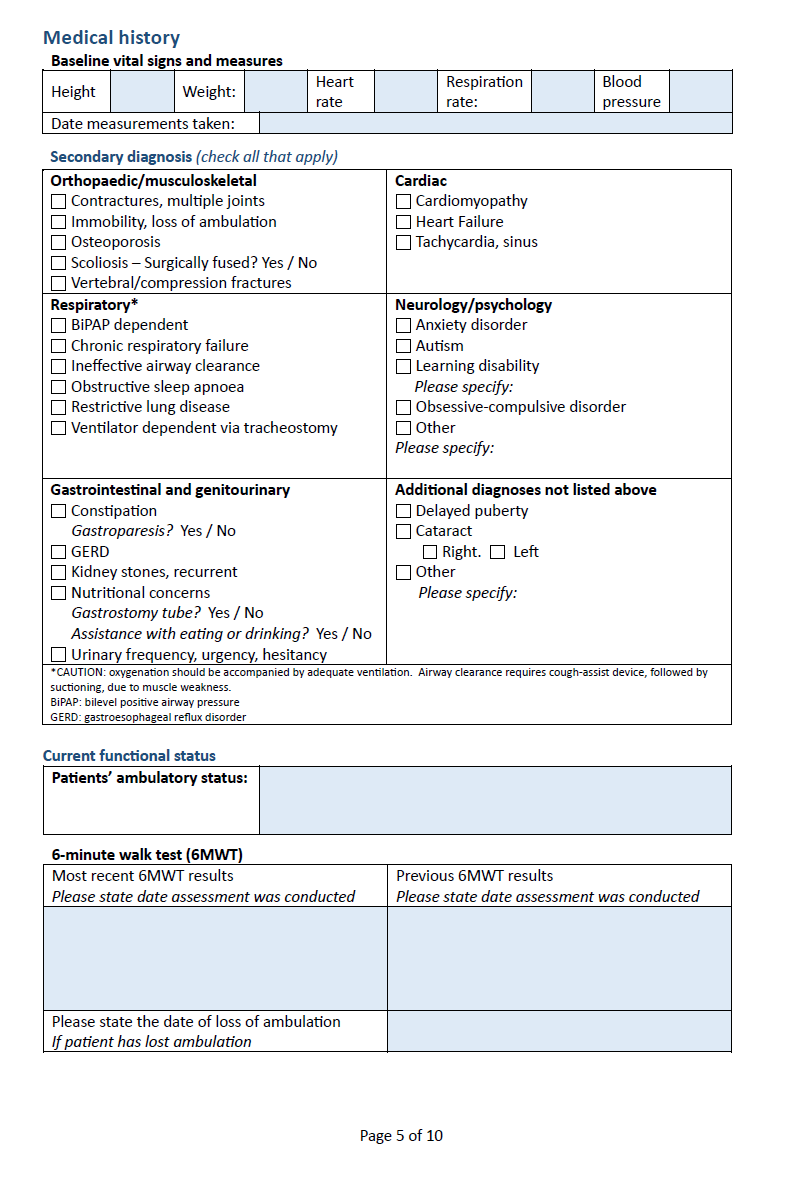


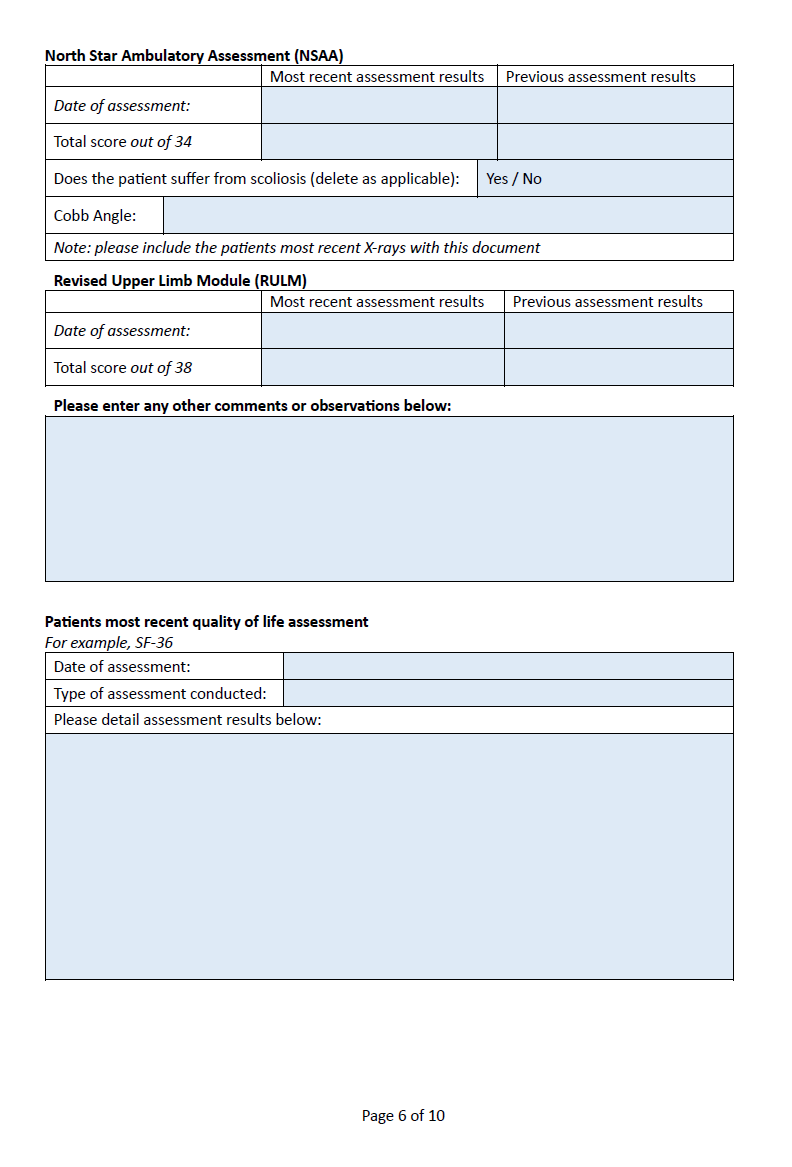


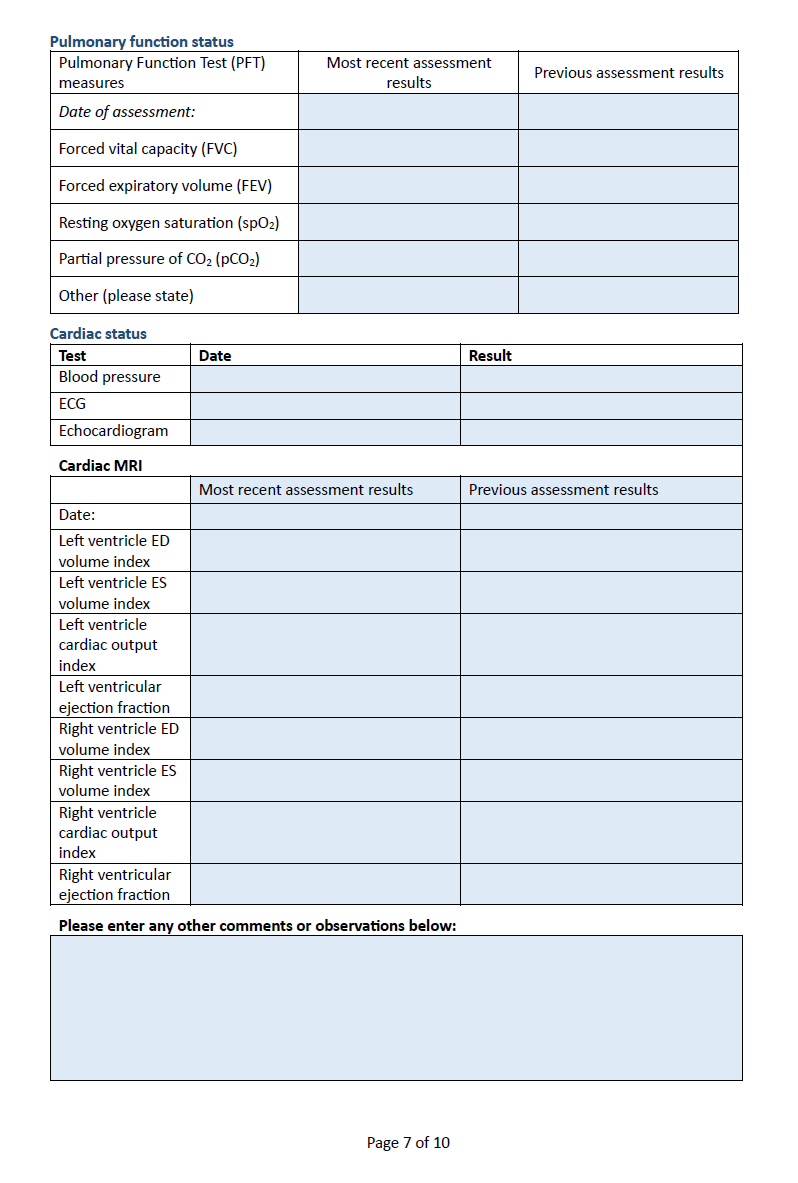


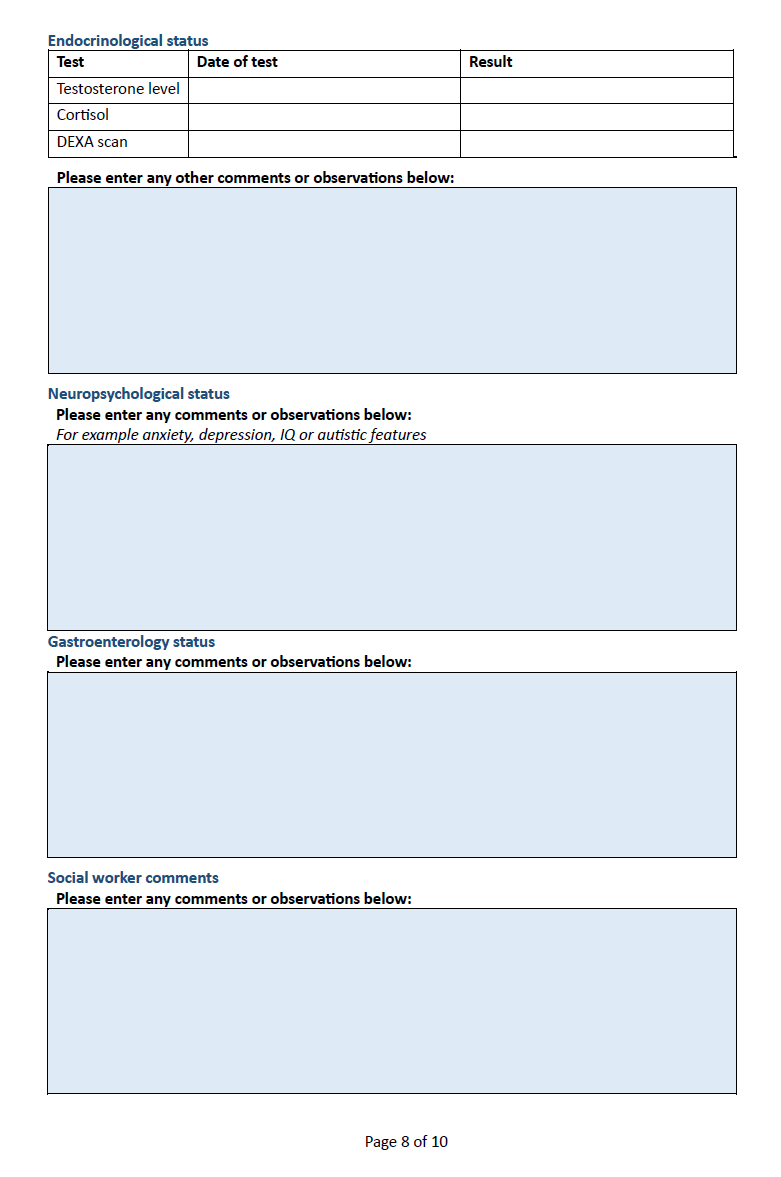


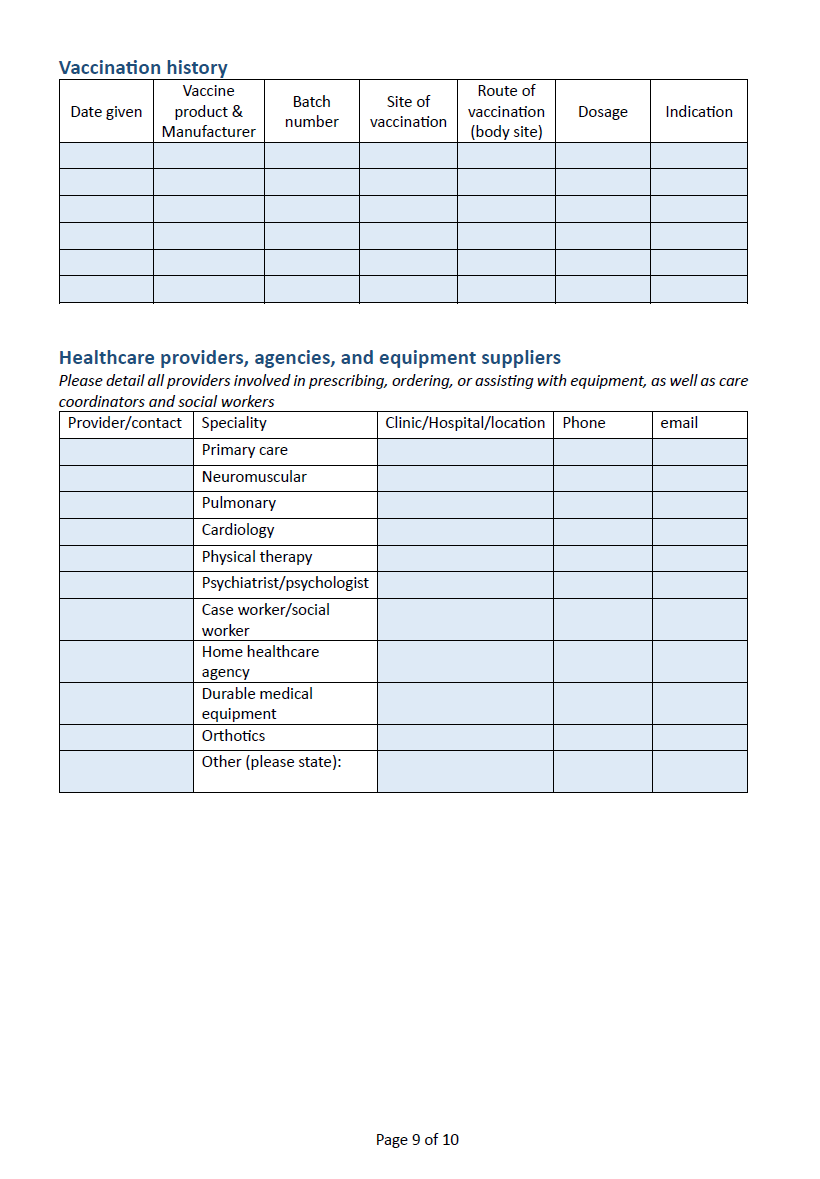


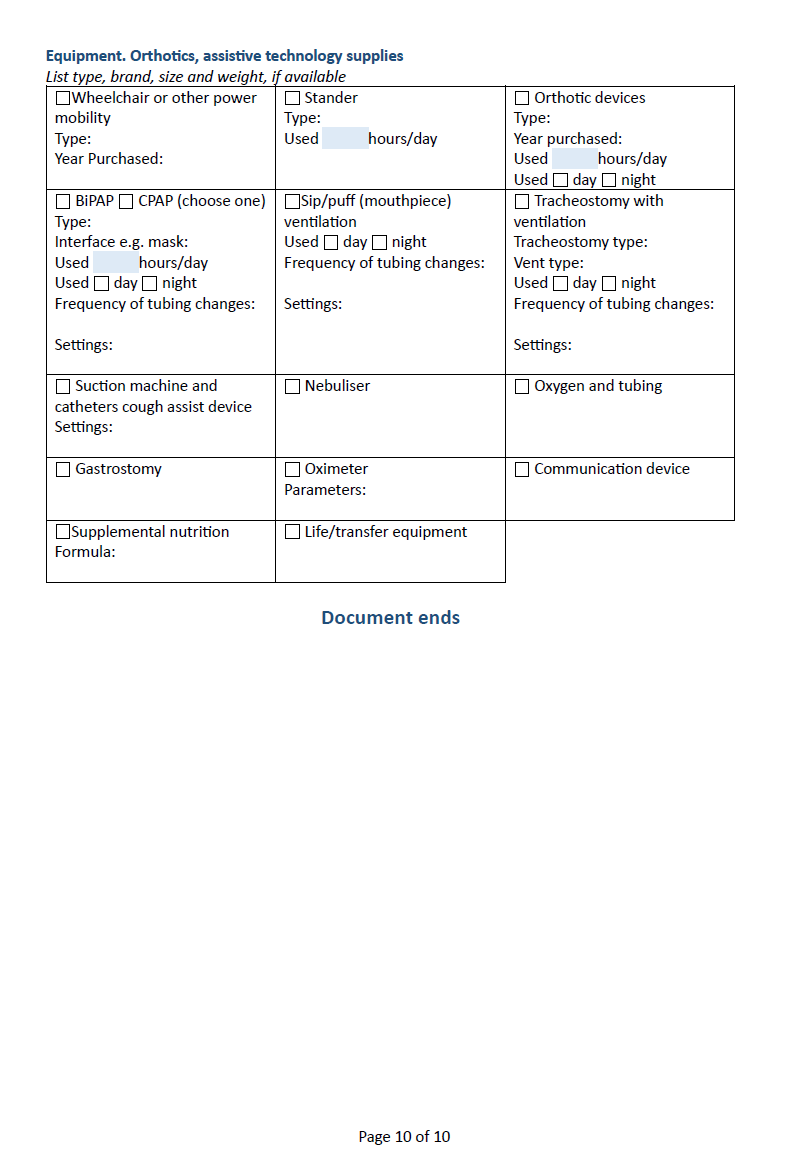

Supplement: Supplementary file 3 — Supplementary Material 3. [file 13023_2024_3270_MOESM3_ESM.docx]
